# The loopy world of cohesin

**DOI:** 10.7554/eLife.71585

**Published:** 2021-07-26

**Authors:** Kazuhiro Maeshima, Shiori Iida

**Affiliations:** 1 Genome Dynamics Laboratory, National Institute of GeneticsMishimaJapan; 2 Department of Genetics, School of Life Sciences, Sokendai (Graduate University for Advanced Studies)MishimaJapan

**Keywords:** cohesin, smc complexes, sister chromatid cohesion, DNA loop extrusion, structural biology, biophysical simulation, Human, *S. cerevisiae*, *S. pombe*

## Abstract

DNA loops can be formed by a mechanism in which the cohesin complex pulls DNA strands through its ring structure using biased Brownian motion.

**Related research article** Higashi TL, Tang M, Pobegalov G, Molodtsov M, Uhlmann F. 2021. A Brownian ratchet model for DNA loop extrusion by the cohesin complex. *eLife* **10**:e67530. doi: 10.7554/eLife.67530

How are very long strands of genomic DNA stored in a tiny cell? In eukaryotes, genomic DNA is wrapped around core histones to form nucleosomes (Olins and Olins, 2003), which are associated with various proteins that package the DNA into chromatin so it can fit inside the nucleus (Maeshima et al., 2021). While some protein complexes are known to play a critical role in the organization of chromatin like loops that allow RNA transcription or DNA replication to be carried out, it remains unclear how chromatin is arranged into such loops.

A protein complex thought to be involved in these arrangements is a ring-like structure called cohesin (Figure 1A). It performs two main roles: firstly, it provides ‘cohesion’ between replicated chromatin by holding sister chromatids together during cell division until the cell is ready to segregate them into two daughter cells (Nasmyth and Haering, 2005; Davidson and Peters, 2021). Secondly, it makes chromatin loops that build functional domains, limitingcurbing chromatin motion (Nozaki et al., 2017; Davidson and Peters, 2021). But how does cohesin carry out these two distinct jobs?

**Figure 1.**
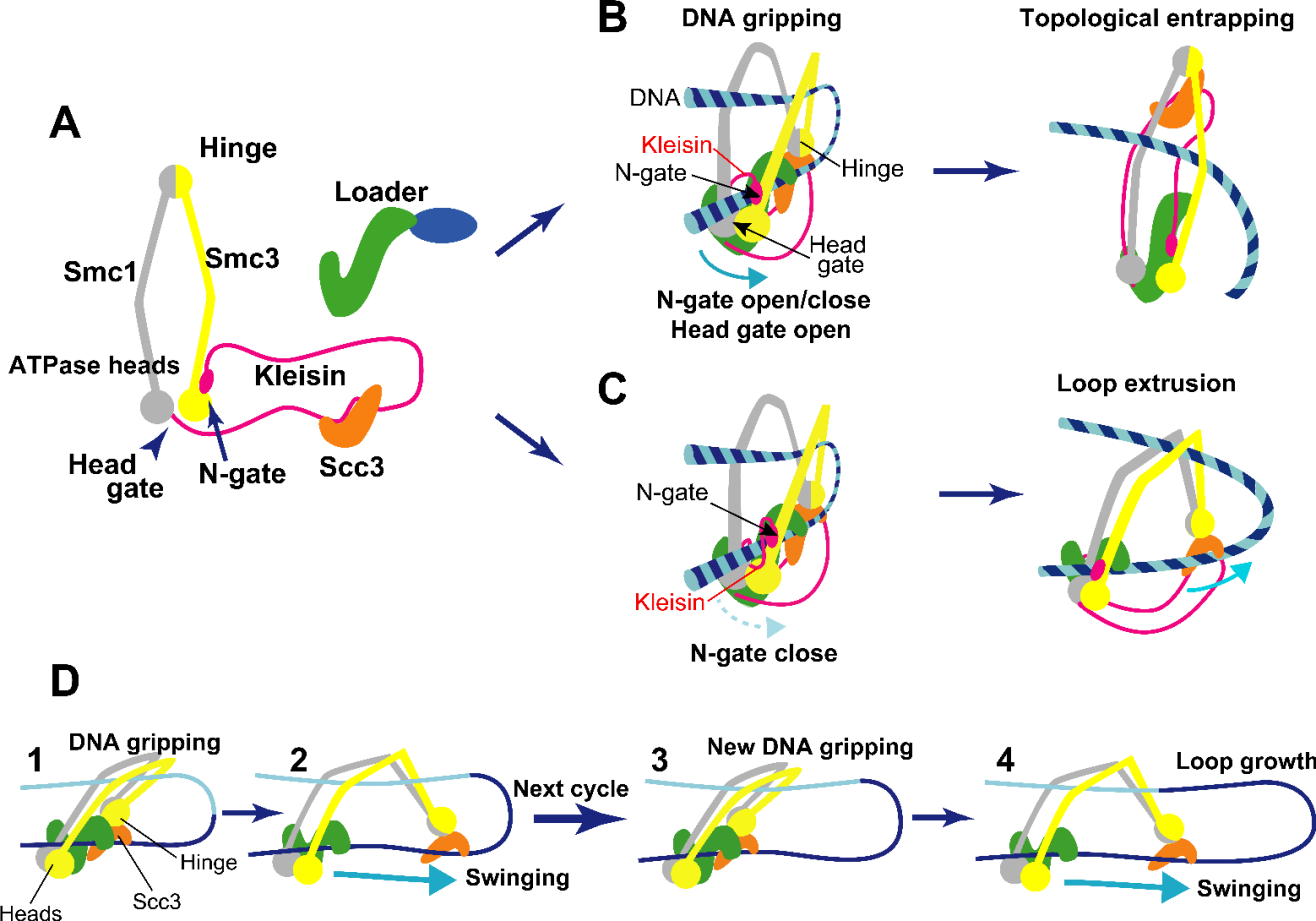
Structure and activity of cohesin. (**A**) Structural overview of the cohesin complex subunits. The two main subunits Smc1/Smc3 (gray and yellow) associate at a hinge on one end and at their ATPase heads on the other. These subunits form a ring-like shape when they are bound by a long flexible kleisin protein (magenta). Kleisin also interacts with additional subunits: the cohesin loader complex (green and blue) and an Scc3 protein (orange). The ATPase heads form a gate that DNA can go through called the head gate, while the interaction between kleisin and the ATPase head of Smc3 forms another gate called the N-gate. (**B**) Topological entrapping: the kleisin N-gate opens, allowing a free strand of DNA to enter it. The N-gate then closes, trapping the DNA strand between the N-gate and the head gate. Once in the DNA gripping position, the head gate is opened by ATP hydrolysis to complete DNA entry. The Smc hinge moves away from the ATPase heads straightening up the cohesin, and leading to topological DNA entrapping. (**C**) Loop extrusion: in this case, a free DNA strand becomes bound by the hinge module and the ATPase heads forming a loop without crossing either gate. Since the kleisin N-gate does not open, even if the head gate is opened by ATP hydrolysis, DNA cannot cross the cohesin ring. Once in this alternative DNA gripping position, Smc hinge, with Scc3 and DNA bound, moves away from the ATPase heads, pulling the DNA with it, and lengthening the loop. (**D**) Schematic for the loop extrusion process. Step 1: in the DNA gripping state, prior to ATP hydrolysis, the two DNA binding modules (hinge and heads) are in close proximity. Step 2: following ATP hydrolysis, the hinge, along with Scc3 bound to DNA, detaches from the head and moves away like a swing. This swinging motion of the hinge module is considered to be driven by diffusion (biased Brownian motion), and pulls the DNA with it. Steps 3 and 4: the next loop extrusion cycle proceeds to promote loop growth when the hinge module returns to form a new gripping state.

A recent model, called loop extrusion, suggests that genomic DNA is constantly pushed out through the cohesin ring to form loops (Davidson and Peters, 2021). This mechanism is thought to keep local regions of DNA together while disentangling them from other parts of the genome. Previous in vitro experiments have shown that cohesin is able to capture protein-free DNA in its ring through a mechanism called topological DNA entrapping or loading (Figure 1B; Murayama and Uhlmann, 2014). More recent research has reported that cohesin can also form loops by extruding sections of DNA not bound to protein (Davidson et al., 2019). However, how cohesin pushes out DNA through its ring structure remained unclear. Now, in eLife, Frank Uhlmann and colleagues from the Francis Crick Institute and University College London – including Torahiko Higashi as first author – report a potential mechanism for how cohesin forms DNA loops in vitro based on cryo-electron microscopy and biochemical observations (Higashi et al., 2021).

Cohesin contains two subunits called Smc1 and Smc3 that each contain a hinge domain that joins the two molecules together (Figure 1A; Davidson and Peters, 2021). At the other end of each subunit is an ATPase head which can bind and hydrolyze ATP: this causes the ATPase head to join together or disassociate from one another depending on the situation. A third protein called kleisin connects to the Smc1 ATPase head via its C-terminal domain, while its N-terminal domain can associate and separate from the Smc3 ATPase head. Kleisin also interacts with two other subunits, the cohesin loader and the Scc3 protein. The cohesin ring structure has two ‘gates’ that DNA can go through: the ‘N-gate’, where the N-terminal of kleisin binds to Smc3, and the ‘head gate’, where the two ATPase head domains of Smc1 and Smc3 meet (Figure 1A).

Previously, a group of researchers – including some of the researchers involved in the Higashi et al. study – described how ATP-dependent structural changes in cohesin drive DNA entry into the ring for topological DNA entrapping (Figure 1B; Higashi et al., 2020). First, the N-gate opens upon ATP binding, and DNA crosses it, occupying the space between kleisin and the two ATPase heads. Then, the N-gate closes as the cohesin loader locks the DNA against the ATPase head gate, and cohesin enters the ‘DNA gripping state’, in which DNA is trapped between the two gates. Finally, the ATP is hydrolyzed, which opens the head gate, straightening up the complex, and the DNA goes through that gate, entering the ring and leading to topological DNA entrapping (Figure 1B).

Higashi et al. then realized that if DNA does not cross the kleisin N-gate, the DNA gripping state could extrude DNA into a loop (Figure 1C; Higashi et al., 2021). Cohesin’s DNA loop extrusion activity can therefore be explained as a ‘branching path’ of the topological DNA entrapping reaction. In this case, when DNA is in the gripping state, it is bound to cohesin in two places: at the Smc hinge (through Scc3) and at the ATPase heads (Figure 1C). Following ATP hydrolysis, these two binding sites (or modules) separate, but because the N-gate is closed, DNA cannot go through the gates to fully enter the ring, like in topological DNA entrapping. Instead it loops into a U shape. The hinge module, attached to DNA via Scc3, swings away from the head module by diffusion (biased Brownian motion)(Figure 1C), extruding DNA (Figure 1D, steps 1 and 2). When the hinge module returns to its original position to form of a new gripping state, the next loop extrusion cycle can proceed, driving loop growth (Figure 1D, steps 3 and 4).

Higashi et al.’s work provides mechanistic insight into the looping activity of cohesin, as well as posing important questions regarding its biological relevance. Firstly, how is the N-gate kept closed in the ‘branching path’ of loop extrusion the topological DNA entrapping? The electrostatic interactions that contribute to keeping the N-gate closed are facilitated by low salt conditions that are not physiological, implying that loop extrusion may be much easier in vitro.

Secondly, is loop extrusion activity conserved in other SMC-related complexes between different organisms? The family of SMC complexes is widely spread from bacteria to human, and loop extrusion activity has been reported using cohesin from both human and fission yeast, and condensin – a SMC complex involved in chromosome assembly during cell division – from budding yeast (Davidson et al., 2019; Higashi et al., 2021; Ganji et al., 2018; Hirano, 2016). Since loop extrusion seems to depend on the distance between the hinge and head modules, certain SMC-related complexes, like SMC5/6 complexes and bacterial Smc complexes, may not allow this activity (Higashi et al., 2021). This suggests that loop extrusion may be unique to specific SMC complexes, and potentially only possible in certain organisms.

Finally, the loop extrusion activity by cohesin and condensin has been only observed in vitro, with protein-free DNA (Davidson et al., 2019; Higashi et al., 2021; Ganji et al., 2018). But in vivo, how do these proteins cope with eukaryotic chromatin, which is bound to histones forming clusters seems to consist of nucleosome clusters with various binding proteins (e.g. Nozaki et al., 2017)? Higashi et al. expect cohesin (or condensin) to be able to bypass single nucleosomes, but not clusters of nucleosomes (Higashi et al., 2021). Therefore, another mechanism may be needed to explain how chromatin loops are formed in living cells. One possibility is diffusion capture in which cohesin (or condensin) stabilizes interactions between binding sites in chromatin (Gerguri et al., 2021).
